# Advances in Brain Imaging Techniques for Patients With Intractable Epilepsy

**DOI:** 10.3389/fnins.2021.699123

**Published:** 2021-08-06

**Authors:** Mubarak Algahtany, Ahmed Abdrabou, Ahmed Elhaddad, Abdulrahman Alghamdi

**Affiliations:** ^1^Division of Neurosurgery, Department of Surgery, College of Medicine, King Khalid University, Abha, Saudi Arabia; ^2^Department of Radiology, Ain Shams University, Cairo, Egypt; ^3^Department of Radiology, Mansoura University, Mansoura, Egypt; ^4^Department of Radiology, Aseer Central Hospital, Abha, Saudi Arabia

**Keywords:** subtle lesion, surgery, brain imaging, epilpesy, advance

## Abstract

Intractable epilepsy, also known as drug resistance or refractory epilepsy, is a major problem affecting nearly one-third of epilepsy patients. Surgical intervention could be an option to treat these patients. Correct identification and localization of epileptogenic foci is a crucial preoperative step. Some of these patients, however, have no abnormality on routine magnetic resonance imaging (MRI) of the brain. Advanced imaging techniques, therefore, can be helpful to identify the area of concern. Moreover, a clear delineation of certain anatomical brain structures and their relation to the surgical lesion or the surgical approach is essential to avoid postoperative complications, and advanced imaging techniques can be very helpful. In this review, we discuss and highlight the use of advanced imaging techniques, particularly positron emission tomography (PET)–MRI, single-photon emission computed tomography, functional MRI, and diffusion tensor imaging–tractography for the preoperative assessment of epileptic patients.

## Introduction

Temporary onset of signs and/or symptoms due to irregular excessive or synchronous neuronal activity in the brain is known as an epileptic seizure (Fisher et al., 2005). Epilepsy is defined as drug-resistant, or intractable, when the two main antiepileptic drugs are not capable of eliminating seizure activity, according to the International League Against Epilepsy (ILAE) (Duncan et al., 2016).

The epileptic focus is the site where the seizure starts (Munari and Bancaud, 1985). Epilepsy surgery aims to identify, remove, or disconnect this focus to achieve seizure-free conditions (Bonelli et al., 2012). The radiological identification of this focus and defining its relations to adjacent critical brain structures, however, could be challenging (Duncan et al., 2016).

For example, one of the most common causes of focal seizures in adults and infants is temporal lobe epilepsy (TLE), which accounts for 30% of all epilepsies (Rastogi et al., 2008). Nearly one-third of patients with TLE develop drug resistance, and in one-third of them, routine magnetic resonance imaging (MRI) of the brain will be normal (Bernardino et al., 2005).

Preoperative workup is required for the evaluation of patients with intractable epilepsy. MRI is the investigation of the choice for regular assessment. An epilepsy-tailored MRI technique was established to detect subtle lesions. This includes three-dimensional (3D) T1-weighted image gradient echo (MPRAGE) and 3D fluid-attenuated inversion recovery (FLAIR) images with isotropic voxels for the detection of focal cortical dysplasia (FCD) and mesial temporal sclerosis (MTS). Coronal and axial high-resolution T2-weighted images with 2–3 mm slice thickness are also used for the same reason. A 3D susceptibility-weighted imaging (SWI) sequence is required for microbleeds and faint calcification. Moreover, coronal T1 inversion recovery is used for better delineation of the gray–white matter interface, 3D T2 if suspected encephalocele, diffusion-weighted imaging (DWI) for detection of ischemic lesion, and post-contrast scan in case of inflammation or tumors. The use of 3T MRI is favored over regular 1.5T MRI machines (Gonçalves Pereira et al., 2006; Coan et al., 2014; Shah and Mittal, 2014).

The most challenging lesions to detect are MTS and FCD. The age of onset of MTS is usually between 4 and 16 years of age, with no gender preference. There may also be a family history of seizures. The diagnosis of MTS requires 3 mm coronal section T2-weighted image or FLAIR, in which gliotic changes appear as hyperintense signals. Atrophy and neuronal loss can also be observed in the T1-weighted image. Surgical intervention in MTS shows a success rate of 90% (Bradley and Shey, 2000; Rastogi et al., 2008; Tsai et al., 2016).

In contrast, FCD is the most common cause of intractable epilepsy in children younger than 3 years of age. It is considered a focal form of malformation of cortical development (MCD) and is thought to be related to an insult during gray matter differentiation. These insults could be ischemic, infectious, toxic, or genetic, and the associated epilepsy is drug-resistant and usually requires surgery (Rastogi et al., 2008).

In many situations, epilepsy-tailored MRI techniques are not sufficient to detect a lesion, and delineate its relations to adjacent eloquent brain structures. Therefore, other advanced techniques, such as positron emission tomography (PET), PET–MRI, functional MRI (fMRI), magnetic resonance spectroscopy (MRS), and diffusion tensor imaging (DTI), can be used. These techniques aim to detect a lesion, its connection, and its intimacy with an eloquent area to limit surgery and avoid post-surgical complications (Knake et al., 2005).

We review different advanced imaging techniques that are useful to detect epilepsy lesions and outline their relations to nearby critical brain structures and highlight the value of these techniques in improving the outcome and safety of epilepsy surgery.

### Positron Emission Tomography

Positron emission tomography is a functional imaging technique that detects glucose uptake and metabolism in various tissues. The most used tracer is 2-deoxy-2[^18^F] fluoro-D-glucose (FDG), which is manufactured in a cyclotron, produces gamma radiation, and can cross the blood–brain barrier. After intravenous injection of FDG, tissue uptake, accumulation, and processing can be detected using a specific PET scanner (Juhász and John, 2020).

**FIGURE 1:**
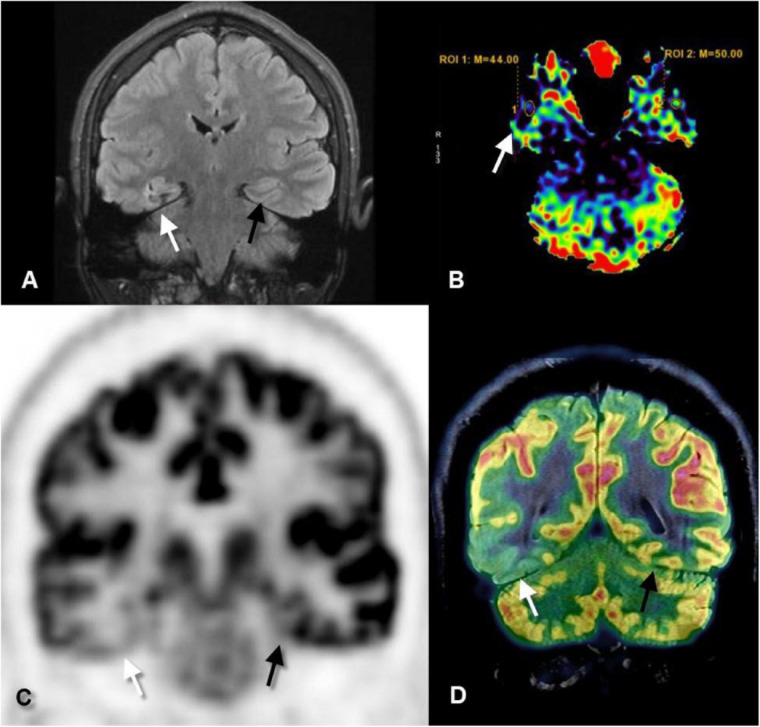
Coronal fluid-attenuated inversion recovery (FLAIR) **(A)**, axial perfusion-weighted imaging-arterial spin-labeled (PWI-ASL) **(B)**, coronal positron emission tomography (PET) **(C)**, and coronal PET–MRI fusion **(D)** images of a 23-year-old male with longstanding epilepsy and underwent the scans in the interictal period. FLAIR image revealed mild increased signal and mild gliotic changes of the right hippocampus (white arrows) as compared to the left one (black arrows). PWI-ASL revealed a decrease in the cerebral blood flow (CBF) in the right hippocampus [region of interest (ROI) 1 = 44] as compared to the left hippocampus (ROI 2 = 50) denoting hypoperfusion. PET and PET–MRI images revealed pronounced hypometabolism of the right temporal lobe particularly the hippocampal formation as compared to the contralateral side. Quantitative data showed standardized uptake value (SUV) of right hippocampus measured 2.56 while on the left hippocampus measured 4.88 (Case courtesy of Prof. Dr. Yasser Abdel Azeem, Ain Shams University).

Positron emission tomography scanning requires a long imaging time (approximately 30 min), so it is suitable to detect epileptic focus, or in another term, the functional deficit zone, during the interictal period. Epileptic focus, which could be a small FCD that is often missed on MRI, appears as an area of low metabolism (reduced uptake) due to low neuronal activity as compared to the rest of the brain tissue (Figure 1). Even if the lesion is detected by conventional MRI, the extent of this lesion is beyond what is visible, and PET can detect the exact damaged area. Therefore, PET provides lateralization and localization information to guide epilepsy surgeons to remove or disconnect only the area of concern while avoiding major surgical procedures and poor postoperative outcomes (Rosenow and Lüders, 2001; Juhász and John, 2020).

Positron emission tomography is superior to conventional MRI in detecting mild malformations of cortical development, which was missed in up to 66% of patients by MRI, while detected in 77% of patients by PET in some of the studies (Kim et al., 2011).

Co-registration of MRI with PET (Figure 1) adds more localizing capability and increases the sensitivity of PET to detect FCD, particularly type I, up to 98% which is commonly missed on routine MRI examination (Salamon et al., 2008). Moreover, surgical resection of the PET–MRI-positive region is associated with better seizure-free outcomes (Lin et al., 2018).

Hybrid PET–MRI has the same sensitivity as PET–CT but has the advantage of less radiation exposure and lower absorbed dose to the brain and eye as the images are acquired in a single session (Oldan et al., 2018). Limitations include high cost, long scan time, and the use of radioactive material which should be prepared in advance (Juhász and John, 2020).

## Single-Photon Emission Computed Tomography

Single-photon emission computed tomography (SPECT) is another nuclear medicine technique that can detect epileptogenic foci during seizure activity. During seizures, blood perfusion to the abnormal area increases up to threefold. SPECT can detect this focal hyperperfusion during ictus; hence, it is called ictal SPECT (Valmier et al., 1989). Technetium-99m hexamethylpropyleneamine oxime (99^*m*^Tc HMPAO) is used as an isotope agent, and a gamma camera is used to detect the radiation emission from the isotope. The injected dose is divided into two halves. The first half is injected during the interictal period, while the second half is injected during seizures, which is detected by an electroencephalograph (EEG) connected to the patient’s scalp. The uptake of the interictal scan is subtracted from the uptake of the ictal scan and the result refers to the focal area of hyperperfusion, and hence the epileptogenic focus (Duncan et al., 2016).

Furthermore, ictal SPECT can be co-registered with MRI, as with PET, and this again increases its accuracy and ability to show better anatomical localization. The new technique is called subtraction ictal SPECT co-registered with MRI (SISCOM). Identifying the epileptogenic focus by SISCOM is associated with good postoperative outcomes and elimination of seizures (Lerner et al., 2009).

SPECT co-registered with MRI is also useful in determining the exact location during placement of intracranial EEG leads for invasive recording that reduces possible complications. Nevertheless, it is sensitive to detect ictal focus in cases of extratemporal lobe epilepsy (Kim et al., 2009).

One limitation of ictal SPECT is the timing of radiotracer injection, which should be within 25 s after onset of ictus to achieve good sensitivity and avoid misleading results (Stamoulis et al., 2017).

## Perfusion MRI

Magnetic resonance imaging can detect changes in blood flow and volume using perfusion techniques. This can be done with intravenous contrast administration, for example, dynamic susceptibility contrast (DSC) or without intravenous contrast administration, for example, arterial spin labeling (ASL). The acquisition time is relatively long (3–4 min), which is why MR perfusion detects hyperperfusion in the early post-ictal period. Perfusion may be performed in the interictal period and the results can be subtracted from the early post-ictal perfusion images which confirmed the epileptogenic focus (Shah and Mittal, 2014; Abud et al., 2015).

In ASL (Figure 1), protons of the arterial blood are labeled by a radiofrequency pulse generated from the MR machine before they enter the cerebral vasculature and exchange with unlabeled protons. ASL can measure the cerebral blood volume (CBV) and cerebral blood flow (CBF) at the area of interest and hence can measure seizure related changes. It is widely used in accompany with conventional MRI as it doesn’t require intravenous contrast injection, it has a low cost, less time, and doesn’t subject the patients to radiation or radioactive hazards. On the contrary, the sensitivity of ASL is affected by the time delay between the onset of seizure and beginning of the scan (Kim et al., 2016).

## Diffusion Tensor Imaging and Fiber Tractography

Diffusion tensor imaging is an advanced MRI technique that monitors the continuous movement of water molecules (Brownian movement). In an isotropic medium, for example, the gray matter, water molecules tend to be distributed randomly with no preferred direction. In an anisotropic medium, for example, white matter fibers, water molecules prefer to diffuse along the long axis of the nerve fibers rather than perpendicular to this axis (preferred direction) (Fujiyoshi et al., 2016; San Martín Molina et al., 2020).

**FIGURE 2:**
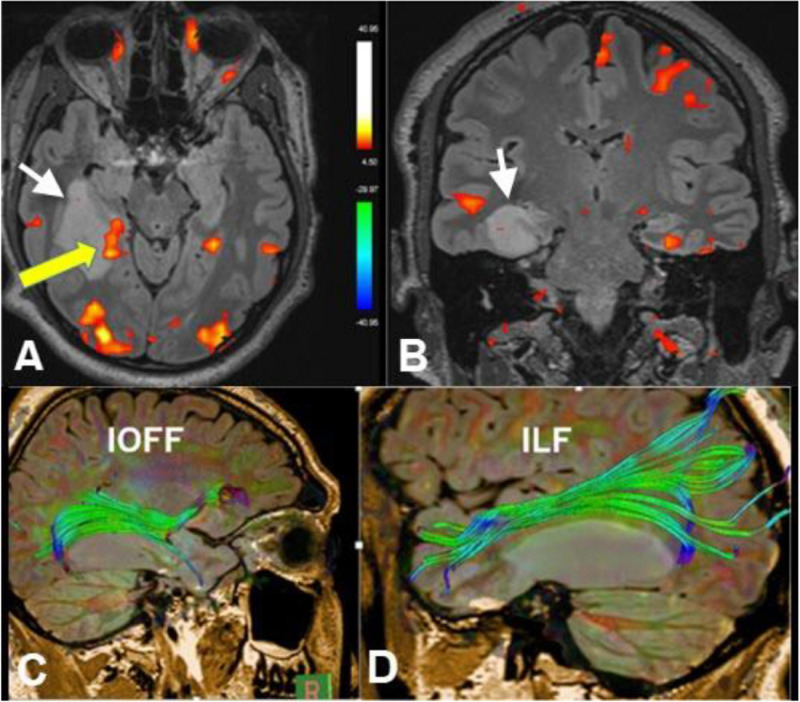
Axial **(A)** and coronal **(B)** functional MRI (fMRI) (memory paradigm), diffusion tensor imaging (DTI)-tractography of the inferior occipitofrontal fasciculus (IOFF) **(C)** and inferior longitudinal fasciculus (ILF) **(D)** of a 30-year-old male with recurrent epilepsy due to right hippocampal low-grade glioma (white arrows). Yellow arrow referred to cortical representation at the posterior aspect of the hippocampus in relation to the lesion after memory activation. Tractography revealed deviation of the IOFF and ILF by the lesion without disruption of the fibers. These data are important in pre-operative planning (Case courtesy of Prof. Dr. Yasser Abdel Azeem, Ain Shams University).

By dividing the matrix into voxels and determining the water movement direction of each voxel, reconstruction of the white matter tracts can be performed by combining voxel directions, which is called tractography (Figure 2). Several tractography algorithms can be used, either deterministic or probabilistic, and the fiber tracts of every white matter bundle can be generated (Rosazza et al., 2018).

**FIGURE 3:**
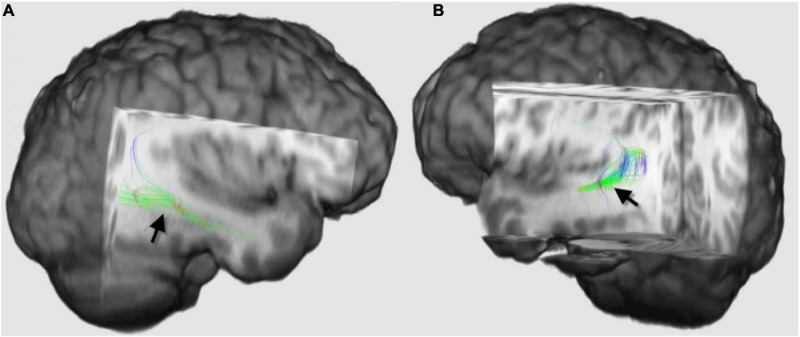
Three-dimensional diffusion tensor imaging (DTI)-tractography images of the Meyer’s loops on the right side **(A)** and left side **(B)** of the same patient (black arrows), which demonstrated how asymmetrical Meyer’s loop could be in the same individual. The loop on the right side projected more into the temporal lobe than the left side. Tractography of this loop and estimation of the distance between the anterior pole of the temporal lobe and these fibers are recommended before anterior temporal lobe resection to guard against postoperative visual field defect.

An important step in preoperative evaluation in patients with intractable epilepsy is to identify Meyer’s loop at the site of surgery. Mayer’s loop is the anterior bundle of optic radiation, which projects variably into the temporal lobe (Figure 3). Anterior temporal lobe resection can cause injury of Meyer’s loop and, consequently, postoperative visual field defects. Identification of Meyer’s loop and measuring the distance from the anterior pole of the temporal lobe is crucial to salvage the bundle during surgery and avoid postoperative complications (Yogarajah et al., 2009; James et al., 2015).

Another white matter bundle that should be preserved during surgery is the arcuate fasciculus. The arcuate fasciculus connects the language areas at the temporal (Wernicke’s) and frontal lobes (Broca’s) and is larger in the dominant hemisphere (Ellmore et al., 2010). Injury or disconnection of this tract during surgery results in conduction aphasia; therefore, DTI-tractography is recommended before frontotemporal resection to preserve this bundle (Li et al., 2013).

## Functional MRI

In the preoperative setting, DTI should be combined with fMRI to estimate the exact risk of surgical intervention. fMRI is a functional imaging technique that is sensitive to changes in oxygen concentration. Activated cortical areas within the brain extract more oxygen from the blood, and its blood oxygen level-dependent (BOLD) signal will change (Figure 2; Gaillard, 2000).

**FIGURE 4:**
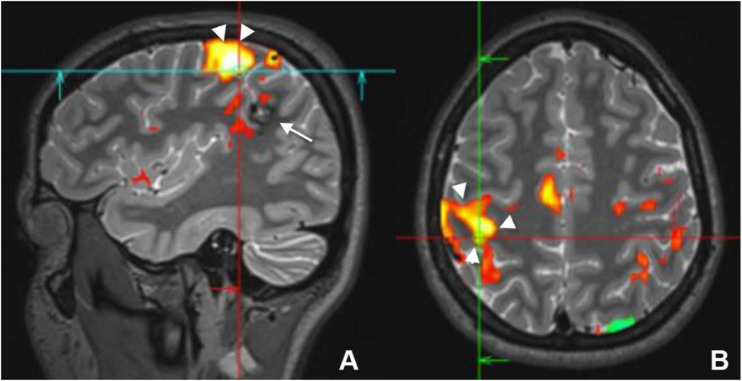
Sagittal **(A)** and axial **(B)** preoperative functional MRI (fMRI) of a young female who had a cavernous hemangioma on the right parietal lobe (white arrow) that caused recurrent seizure. Activation of the motor cortex (white arrowheads) by finger tapping test revealed that the lesion was separable from the motor cortex and it could be resected without postoperative motor deficit.

Each cortical area requires a specific task and a tailored paradigm for its activation and assessment. Activation of the cortical area will result in focal hyperemia and a transient increase in oxygenic consumption (Figures 2, 4), which will consequently cause a detectable increase in the T2^∗^ signal in this region (Yerys et al., 2009).

Evaluation of language function and its side of dominance is essential for safe epilepsy surgery and requires the assessment of receptive (Wernicke) and expressive (Broca’s) language areas. In most healthy right-handed individuals, the left hemisphere is dominant, while in patients with TLE, this pattern is changed; there is a higher incidence of bilateral and right hemisphere dominance (Rosazza et al., 2013). Lateralization of the dominant hemisphere for language was previously carried out by the intracarotid amytal injection (Wada) test or electrocortical stimulation mapping, which are invasive techniques. Consequently, fMRI has been increasingly used to replace such invasive techniques (de Ribaupierre et al., 2012).

Several tasks are required to assess the comprehensive and productive capability of language areas. These include the verbal generation task, which is an easy task that stimulates both frontal and temporal language areas, semantic comprehension tasks that require naming objects and result in activation of temporal areas as well as verbal fluency tasks that activate the frontal more than the temporal regions (Vitali et al., 2011).

Localizing the motor area before surgery is an important step to avoid post-surgical paralysis. Localization of the dominant hemisphere is important before hemispherectomy, and the assessment of the closeness of the epileptogenic focus to the motor area is crucial. Tasks that involve stimulating the foot, finger, and mouth can be used to assess the motor cortex (Figure 4). In children less than 2 years of age and in sedated patients, activation of the motor cortex can be successfully performed using passive movement (Choudhri et al., 2015).

Resting-state fMRI (rs-fMRI) is another emerging technique that does not require a specific task for activation. It detects spontaneous neural activity, which creates fluctuations in the BOLD signal. Some studies have revealed that patients with epilepsy have an abnormal pattern of the resting-state network including decreased connectivity in comparison to the healthy group (Pizoli et al., 2011; Roland et al., 2017).

Although fMRI has a lower risk, lower cost, and more functional localization capability than Wada test, yet it still has some drawbacks. For example, there is no standard threshold to differentiate between activated and non-activated areas, the complexity of some tasks like language and memory requires high level of cooperation between staff and participants and the lack of ideal paradigms for the assessment of some tasks such as memory (Limotai and Mirsattari, 2012; Szaflarski et al., 2017).

## Conclusion

Surgery can be a good treatment option for medically refractory epilepsy. The surgical success may depend on the use of advanced brain imaging techniques to identify the epileptic focus. We recommend the use of multiparametric imaging like conventional MRI with the use of perfusion, SPECT, PET–MRI to increase the chance of correct identification of the epileptogenic focus and the use of fMRI as well as DTI before surgical intervention to map the eloquent cortical areas, such as the language and motor cortex, as well as the connecting white matter tracts, to avoid accidental damage and faulty disconnection during surgery.

## Author Contributions

MA: conceptualization, writing review and editing, and supervision. AAb, AE, and AAl: writing review and editing. All authors contributed to the article and approved the submitted version.

## Conflict of Interest

The authors declare that the research was conducted in the absence of any commercial or financial relationships that could be construed as a potential conflict of interest.

## Publisher’s Note

All claims expressed in this article are solely those of the authors and do not necessarily represent those of their affiliated organizations, or those of the publisher, the editors and the reviewers. Any product that may be evaluated in this article, or claim that may be made by its manufacturer, is not guaranteed or endorsed by the publisher.
